# Optimization and Characterization of a *Galleria mellonella* Larval Infection Model for Virulence Studies and the Evaluation of Therapeutics Against *Streptococcus pneumoniae*

**DOI:** 10.3389/fmicb.2019.00311

**Published:** 2019-02-21

**Authors:** Freya Cools, Eveline Torfs, Juliana Aizawa, Bieke Vanhoutte, Louis Maes, Guy Caljon, Peter Delputte, Davie Cappoen, Paul Cos

**Affiliations:** Laboratory of Microbiology, Parasitology and Hygiene, Department of Pharmaceutical Sciences, University of Antwerp, Wilrijk, Belgium

**Keywords:** *Streptococcus pneumoniae*, *Galleria mellonella*, infection model, virulence, antibiotics, wax moth larvae

## Abstract

*Streptococcus pneumoniae* is the leading cause of bacterial pneumonia. Infection is linked to high morbidity and mortality rates and antibiotic resistance within this pathogen is on the rise. Therefore, there is a need for novel antimicrobial therapies. To lower the time and costs of the drug discovery process, alternative *in vivo* models should be considered. As such, *Galleria mellonella* larvae can be of great value. The larval immunity consisting of several types of haemocytes is remarkably similar to the human innate immune system. Furthermore, these larvae don’t require specific housing, are cheap and are easy to handle. In this study, the use of a *G. mellonella* infection model to study early pneumococcal infections and treatment is proposed. Firstly, the fitness of this model to study pneumococcal virulence factors is confirmed using streptococcal strains TIGR4, ATCC^®^49619, D39 and its capsule-deficient counterpart R6 at different inoculum sizes. The streptococcal polysaccharide capsule is considered the most important virulence factor without which streptococci are unable to sustain an *in vivo* infection. Kaplan–Meier survival curves showed indeed a higher larval survival after infection with streptococcal strain R6 compared to strain D39. Then, the infection was characterized by determining the number of haemocytes, production of oxygen free radicals and bacterial burden at several time points during the course of infection. Lastly, treatment of infected larvae with the standard antibiotics amoxicillin and moxifloxacin was evaluated. Treatment has proven to have a positive outcome on the course of infection, depending on the administered dosage. These data imply that *G. mellonella* larvae can be used to evaluate antimicrobial therapies against *S. pneumoniae*, apart from using the larval model to study streptococcal properties. The in-depth knowledge acquired regarding this model, makes it more suitable for use in future research.

## Introduction

*Streptococcus pneumoniae*, also known as pneumococcus, is the most common cause of bacterial pneumonia, and can cause otitis media and meningitis in children, the elderly and immunocompromised patients. In a 2013 European study, an increase in the incidence of streptococcal bacteremia was reported. According to this study, *S. pneumoniae* was responsible for 11% of all bacteremia reports (de Kraker et al., 2013). In a 2018 European study, the incidence of pneumococcal community acquired pneumonia was estimated to be as high as 166 per 100,000 in Spain in elderly patients, with the incidence of invasive pneumococcal disease (IPD) to be 60 per 100,000. For all-cause pneumonia, the numbers rise to 7,000 per 100,000 in some countries (Torres et al., 2018). Overall, streptococcal infections are linked to high morbidity and mortality rates, even with the introduction of several vaccines. Antibiotic resistance highly depends on the antibiotic class and geographical region, reaching over 80% penicillin resistance in some parts of the African and Asian continents (Rennie, 2012).

While there is an urgent need for new antimicrobial therapies to combat streptococcal infections, this process is very time consuming and costly. One of the steps in this process is testing the potential novel compounds in *in vivo* models. Currently, rodents are the most commonly used animal models. However, these vertebrate models are often expensive, time consuming to maintain and ethically challenging (Graham and Prescott, 2015). Therefore, there is a need for new *in vivo* models for future research that can replace—or in any case significantly lower the amount of—vertebrates used in scientific research by bridging the gap between less complex *in vitro* models and vertebrate *in vivo* models. Over the past years, several alternative *in vivo* models have been proposed.

*Caenorhabditis elegans* is often used as a model due to its low cost, simple growth conditions and short generation time. Also, is has a completely sequenced genome and its immune system shows similarities with the human innate immune system (Schulenburg and Ewbank, 2007; López Hernández et al., 2015). However, this model poses limitations as well. The nematodes can only survive between 15 and 25°C, while human pathogens live at body temperatures. Also, its usefulness is limited by the lack of an adaptive immune system and of intracellular replication of pathogens (Squiban and Kurz, 2011). Adult zebrafish (*Danio rerio*) can also be used to study infectious diseases involving both the innate and adaptive immune system, while in embryos only the innate immunity is present. The embryos are transparent during the first days after fertilization, making it possible to follow an infection in real-time using fluorescent pathogens (Duggan and Mostowy, 2018). Also for this organism, the full genome is available (Chakraborty et al., 2009; Meijer and Spaink, 2011; López Hernández et al., 2015). Zebrafish can only survive at about 28°C, which limits their use for the study of human pathogens (Meeker and Trede, 2008).

*Galleria mellonella* larvae are larvae of the greater wax moth with a length of approx. 20 mm. These larvae can be obtained at commercial angler’s stores. Currently, there is only one company providing lab-grade larvae and genotyping has recently been carried out (Champion et al., 2016; Lange et al., 2018). Overall, *G. mellonella* larvae are cheap, do not require any specific caging and are easy to handle. Therefore, costs of this model are significantly lower compared to other ones (López Hernández et al., 2015; Tsai et al., 2016). Additionally, larvae can be maintained at 37°C, holding a big advantage over the *C. elegans* model when studying human pathogens (Loh et al., 2013; Champion et al., 2016). Wax moth larvae do not possess an adaptive immune system, but they do have an immune system analogous to the human innate immune system, making this model suitable to study early host-pathogen interactions and infections not involving adaptive immune responses. The haemolymph of these larvae consists of at least 8 types of haemocytes, of which plasmatocytes and granular cells possess macrophage-like functionalities, being involved in phagocytosis and encapsulation (Tsai et al., 2016). *G. mellonella* larvae have been used in previous research to study fungal and bacterial infections. It has been shown to be possible to study the virulence of different *Candida albicans* strains, and also to evaluate antifungal compounds and study biofilm formation in this model (Dunphy et al., 2003; Borghi et al., 2014; Borowiecki et al., 2018). For bacterial infections, it has been possible to infect larvae with *Listeria monocytogenes* and *Legionella pneumophila*. Also antimicrobial therapies against *Pseudomonas aeruginosa* have already been tested (Joyce and Gahan, 2010; Harding et al., 2013; Hill et al., 2014).

In this study, we propose the use of a *G. mellonella* infection model to study early pneumococcal infections and treatment. We determined the pathogenicity of several *S. pneumoniae* serotypes and thereby confirmed the suitability of this infection model (Evans and Rozen, 2012). Additionally, we further characterized the infection by determining the number of haemocytes, production of oxygen free radicals and bacterial burden in the haemolymph at several time points during the course of infection. Overall, our data demonstrate that *G. mellonella* larvae can be used to evaluate antimicrobial therapies for *S. pneumoniae* and give rise to more in-depth knowledge regarding this model, making it more suitable for use in future research.

## Materials and Methods

### *G. mellonella* Larvae and Bacterial Strains

*G. mellonella* last-instar larvae were purchased from a local vendor (Anaconda Reptiles, Kontich) and stored in wood chips at 15°C before use. Four hours before use, larvae were put at 4°C. Only healthy-looking larvae with no signs of melanization were used in the experiments. *S. pneumoniae* strains are listed in Table 1. Bacteria were cultured in brain-heart infusion (BHI) broth (LabM) or on 5% sheep blood agar plates (Tryptic Soy Agar from LabM, defibrinated sheep blood from Oxoid) at 37°C and 5% CO_2_. Agar plates or broth were supplemented with 1 μg/mL tetracycline (Sigma) when required. To obtain heat-killed streptococci, TIGR4 bacteria grown to mid-log phase were incubated for 30 min at 56°C. Killing was confirmed by plating out the suspension. To allow bacterial enumeration from *G. mellonella* haemolymph, streptococcal mutant TIGR4/10 (tetracycline resistant) was obtained through integration of plasmid pKB01_tagRFP into the streptococcal genome, as described previously. pKB01_tagRFP was a gift from Jan-Willem Veening (Addgene plasmid #69103) (Martin et al., 1995; Beilharz et al., 2015). Briefly, plasmid pKB01_tagRFP was purified using a NucleoSpin Plasmid EasyPure kit (Macherey-Nagel) and transformed into *S. pneumoniae* TIGR4 using competence-stimulating peptide 2 (CSP-2). Bacteria were grown in CAT-medium [1.0% casitone (BD Biosciences), 0.5% tryptone (Sigma-Aldrich), 0.1% yeast extract (Sigma-Aldrich), 0.5% NaCl (Merck), 0.2% glucose (Sigma-Aldrich), 16.7 M K_2_HPO_4_ (Roth)] until an OD_600nm_ of 0.5 was reached. Bacteria were diluted 50 times in CTM-1 medium [CAT-medium supplemented with 1 mM CaCl_2_ (Merck), 0.2% bovine serum albumin (Sigma-Aldrich), pH 7.2] until OD_600nm_ 0.15. Bacteria were then concentrated 10 times in CTM-1 medium and 15% glycerol was added. Bacteria were kept at –80°C until use. When needed, bacteria were thawed on ice and diluted 10 times in CTM-2 medium (CTM-1 medium, pH 7.9). 2% (v/v) 500 μg/mL CSP-2 stock was added to 200 μL aliquots and bacteria were incubated for 20 min at 37°C. Plasmid DNA was added and bacteria were incubated again for 20 min at 30°C to allow the uptake of the DNA. Bacteria were plated out on blood agar plates containing 1 μg/mL tetracycline and incubated overnight at 37°C and 5% CO_2_.

**Table 1:** *S. pneumoniae* strains used in this study.

Strain	Serotype	Source
TIGR4	Serotype 4	ATCC^®^BAA-334
D39	Serotype 2	NCTC^®^7466
R6	Serotype 2^-^	NCTC^®^13276
ATCC 49619	Serotype 19F	ATCC^®^49619
TIGR4/10	Serotype 4, Tet^R^	Derived from TIGR4, constructed with Addgene plasmid #69103


### *G. mellonella* Killing Assay

A sterile 20 μL Hamilton syringe was used to inject 10 μL aliquots of bacterial suspensions in PBS into the hindmost left proleg of *G. mellonella*. Larvae were infected with 10^4^, 10^5^, or 10^6^ CFU/larvae, with a minimum of 15 larvae per group. The control group was injected with 10 μL phosphate buffered saline (PBS) (Life Technologies). Following the injections, larvae were incubated at 37°C in the dark for several days to allow the progression of the pneumococcal infection. Every 24 h, larvae were scored as dead or alive. Larvae were determined dead when no signs of movement could be observed in response to external stimuli and when the larvae showed dark pigmentation due to melanization. For each strain, 4 independent repeats were carried out.

### *G. mellonella* Bacterial Burden

To determine the bacterial burden, larvae were infected with streptococcal strain TIGR4/10, with a minimum of 15 larvae per time point. At fixed time points after infection (1, 24, 48, and 72 h p.i.), alive larvae were bled into ice-cold microcentrifuge tubes and haemolymph of 5 larvae was pooled together and further processed as one sample. Bacterial burden was determined using the viable plate count method with blood agar plates containing 1 μg/mL tetracycline to inhibit growth of native larval flora and allow streptococcal selection. For each time point, 5 independent repeats were carried out, with preferably two pooled samples per repeat.

### *G. mellonella* Treatment Assay

Larvae infected with streptococcal strain TIGR4/10, D39, or ATCC 49619 at a concentration of 10^6^ CFU/larvae were treated with 10 μL amoxicillin (Sigma-Aldrich), moxifloxacin (Fluka) or tetracycline (Sigma-Alrich) 1 h p.i. at different concentrations (0.1, 1, or 10 μg/mL for amoxicillin and tetracycline and 1, 10, or 100 μg/mL for moxifloxacin) through injection into the hindmost right proleg. After treatment, larvae were incubated and scored as dead or alive as described previously. For each strain, at least 3 independent repeats were carried out with a minimum of 10 larvae per group. To determine the bacterial load in the haemolymph after treatment, bacterial burden was determined as described above. For each time point, 3 independent repeats were carried out with two samples per repeat.

### Preparation and Counting of Larval Haemocytes

At fixed time points p.i. (1, 24, 48, and 72 h p.i.), alive larvae infected with several streptococcal strains were disinfected using 70% ethanol after which they were bled into ice-cold microcentrifuge tubes containing 50 μL of insect physiological solution (IPS) [150 mM NaCl, 5 mM KCl (Sigma-Aldrich), 10 mM tris HCl (Sigma-Aldrich), pH 6.9] containing 30 mM EDTA (Sigma-Aldrich) and 30 mM sodium citrate (VWR) as anticoagulants. Haemolymph of 5 larvae was pooled together into 1 tube and further processed as one sample. Cells were diluted ten times in PBS and counted using KOVA^®^Glasstic^®^Slide 10 counting chambers (Thermo Fisher Scientific). For each time point, 4 independent repeats were carried out, with preferably two pooled samples per repeat.

### Detection of Oxygen Free Radicals

Electron paramagnetic resonance spectroscopy (EPR) was used to measure the formation of oxygen free radicals. At different time points p.i. (1, 24, 48, and 72 h p.i.), alive larvae were bled out into ice-cold microcentrifuge tubes. Haemolymph of 5 larvae was pooled together and further processed as one sample. The haemolymph was diluted 100 × in 1 mL Krebs-Hepes Buffer (KHB, pH 7.4) before adding 1-hydroxy-3-methoxycarbonyl-2,2,5,5-tetramethylpyrrolidine (CM-H) as a spin probe. After incubation for 90 min at 37°C, 50 μL of the suspension was sampled into a capillary tube for measurement. All EPR measurements were performed on a Magnettech MiniScope MS 200 spectrometer as follows: frequency, 9.4 GHz; power: 5 dBm (3.16 mW); modulation frequency, 100 kHz; modulation amplitude, 0.1 mT; sweep time, 30 s; time constant, 0.1; sweep width, 10 mT; and number of scans, 1. For the measurements, the analyzed samples (50 μl) were contained in glass capillaries (Hirschmann). Data were analyzed using the SpecView software. Peak height was measured and reported in arbitrary units (a.u.). For each time point, at least 4 independent repeats were carried out, with preferably two pooled samples per repeat.

### Statistical Analyses

Data were analyzed for statistical significance using GraphPad Prism Version 7. Continuous variables were compared by two-way Anova, unpaired *t*-test or survival analysis. Statistical significance was defined as *p* < 0.05. Statistical significance between two groups is visualized as asterisks in figures (^∗^*p* ≤ 0.05; ^∗∗^*p* ≤ 0.01; ^∗∗∗^*p* ≤ 0.001; ^∗∗∗∗^*p* ≤ 0.0001).

## Results

### Survival of Larvae Was Dependent on MOI, Serotype, and Presence of Virulence Factors

**FIGURE 1:**
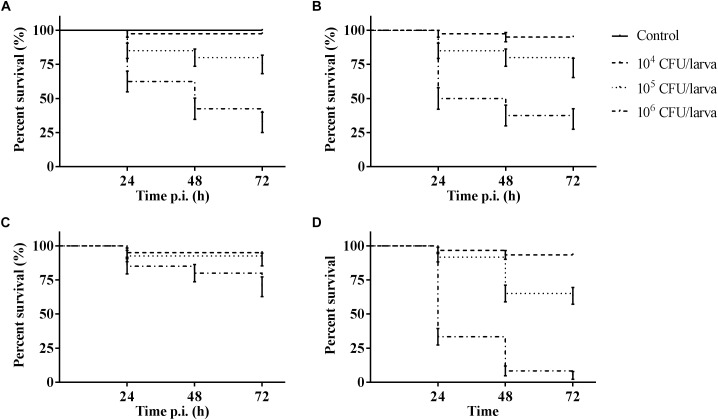
Kaplan–Meier survival curves of *G. mellonella* larvae after infection with *S. pneumoniae* at different inoculum sizes (10^4^ CFU/larva, 10^5^ CFU/larva and 10^6^ CFU/larva). **(A)** Infection with strain TIGR4; **(B)** Infection with strain D39; **(C)** Infection with strain R6; **(D)** Infection with strain ATCC 49619. Control group is injected with PBS. Error bars represent SE (standard error) (*n* = 15 **×** 4).

The outcome of infection in the *G. mellonella* model was – as it is in vertebrate models – dependent on both MOI (TIGR4 *p* < 0.0001; R6 *p* = 0.0044; D39 *p* < 0.0001; ATCC 49619 *p* < 0.0001; Survival analysis, Log-rank Mantel-Cox test) and streptococcal strain (Figure 1). For all strains, administering 10^4^ CFU/larvae hardly led to any larval death, while the effect of administering 10^6^ CFU/larvae was variable. For strains D39 and TIGR4 – which differ in serotype – there was no difference in virulence (*p* = 0.8961; Survival analysis, Log-rank Mantel-Cox test). It should be noted that, while there was no difference in larval survival, the visual pathology of the larvae infected with strain D39 seemed worse than that of strain TIGR4. The melanization process in larvae infected with strain D39 occurred both faster and more intense and their mobility steadily decreased. For larvae infected with strain TIGR4, a more diverse result was observed. Despite the similarity in survival curves, strain D39 was considered more virulent compared to strain TIGR4. Polysaccharide capsule deficient strain R6, which originates from strain D39, showed a clearly attenuated virulence compared to strain D39 (*p* = 0.0008; Survival analysis, Log-rank Mantel-Cox test). The polysaccharide capsule is known as one of the most important virulence factors, which is in accordance with the loss of virulence in this model when the capsule is not present (Kadioglu et al., 2008; Preston and Dockrell, 2008). While strains TIGR4 and D39 showed no difference in survival, there was a significant difference between those strains and strain ATCC 49619, which proved to be the most virulent (D39 and ATCC 49619: *p* = 0.0006; TIGR4 and ATCC 49619: *p* < 0.0001; Survival analysis, Log-rank Mantel-Cox test).

### Bacterial Burden Decreased Over Time

**FIGURE 2:**
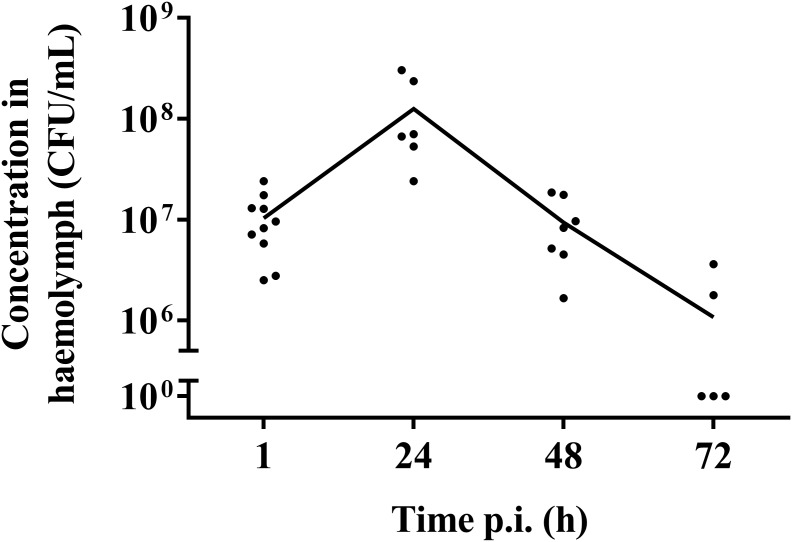
Streptococcal burden in a *G. mellonella* infection model using streptococcal strain TIGR4/10. After a rapid bacterial multiplication (1–24 h), larvae that survived showed a decrease in bacterial burden over the course of the infection (48–72 h). The mean is shown (*n* = 2 × 5).

One hour post-infection, larval burden of *S. pneumoniae* strain TIGR4 decreased with 1 log compared to the initial inoculum of (1.37 ± 0.64) ^∗^ 10^8^ CFU/mL to (1.04 ± 0.07) ^∗^ 10^7^ CFU/mL (Figure 2). After 24 h, the burden increased to (1.26 ± 0.2) ^∗^ 10^8^ CFU/mL 24 h p.i. Larvae that outlived the infection over 24 h, seemed to start clearing the streptococci [(9.38 ± 0.93) ^∗^ 10^6^ CFU/mL 48 h p.i.; (1.08 ± 0.33) ^∗^ 10^6^ CFU/mL 72 h p.i.]. This is probably due to the efficient phagocytosis mechanism of larval haemocytes (Kavanagh and Reeves, 2004; Wojda, 2017). It should be noted that 72 h post-infection, the presence of streptococci in haemolymph could no longer be determined for 60% of all samples. Of note, there was no difference in survival between infection with strain TIGR4 and tetracycline-resistant strain TIGR4/10 (Supplementary Figure 1).

### Number of Haemocytes Did Not Differ Over Time

**FIGURE 3:**
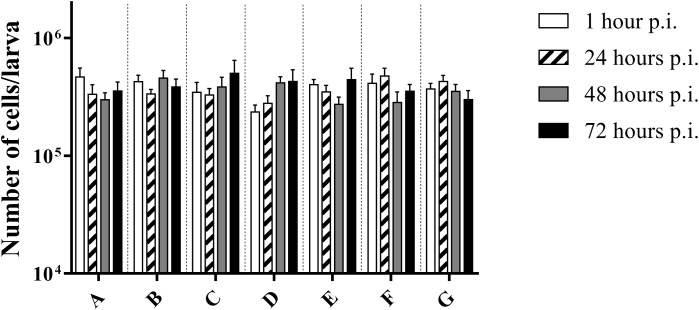
Number of cells in haemolymph of *G. mellonella* at different time points post-infection. **(A)** Non-injected control group; **(B)** PBS-injected control group; **(C)** Infection with heat-killed bacteria; **(D)** Infection strain TIGR4; **(E)** Infection with strain ATCC 49619; **(F)** Infection with strain D39; **(G)** Infection with strain R6. Error bars represent SE (standard error). There are no statistical differences between infected and control groups (Two-way Anova, *p* < 0.05) (*n* = 2 × 4).

There was no difference in the number of cells between the control group and infected larvae (*p* = 0.3940; two-way Anova) (Figure 3). This suggests that there was no influx of haemocytes that can be accredited to the bacterial infection, nor do haemocytes of infected larvae go into apoptosis or necrosis more quickly. Overall, an average of (3.72 ± 0.68) ^∗^ 10^5^ cells/larvae was counted.

### Production of Oxygen Free Radicals Differed During the Course of Infection

**FIGURE 4:**
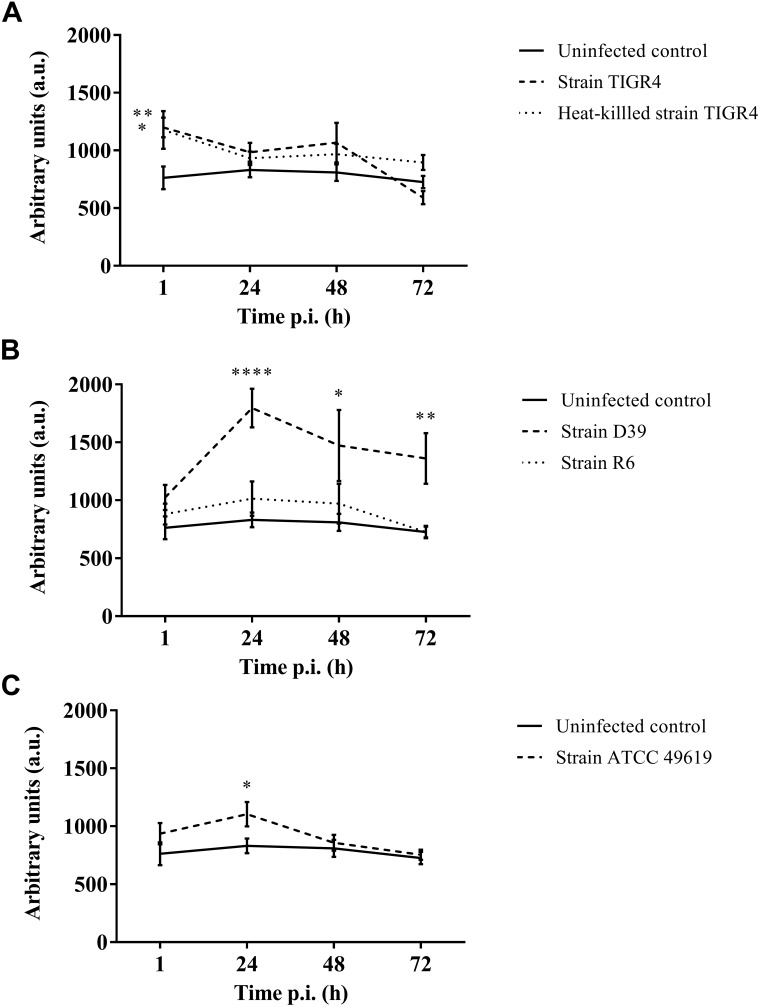
Production of oxygen free radicals of haemocytes of *G. mellonella* at different time points post-infection. Amount of oxygen radicals captured by CM-H is calculated by measuring the peak height after 90 min and expressed in arbitrary units. **(A)** Infection with Strain TIGR4 and heat-killed bacteria; **(B)** Infection with strain D39 and R6; **(C)** Infection with strain ATCC 49619. Error bars represent SE (standard error). Asterisks represent statistical differences between infected groups and the uninfected control group (Unpaired *t*-test, *p* ≤ 0.05, ^∗^*p* ≤ 0.05, ^∗∗^*p* ≤ 0.01, ^∗∗∗∗^*p* ≤ 0.0001) (*n* = 2 **×** 4).

Haemocytes are known to produce oxygen free radicals as a way of battling infections. This can be monitored using the spin probe CM-H (Slepneva et al., 1999). Directly after infection, an increase in production of oxygen free radicals could be observed between the control group and infected larvae for strains TIGR4 (*p* = 0.0100, Unpaired *t*-test) (Figure 4A). Afterward, there was no detectable difference between this strain and the control group (24 h p.i. *p* = 0.1731; 48 h p.i. *p* = 01314; 72 h p.i. *p* = 0.1517; Unpaired *t*-test). This concurs with previous results (Figure 2), where also the streptococcal burden was lowered after 24 h, presuming the infection was being cleared.

Furthermore, for strain D39 (Figure 4B), a clear increase in oxygen free radicals could be observed during the course of infection compared to the control group (1 h p.i. *p* = 0.1067; 24 h p.i. *p* < 0.0001; 48 h p.i. *p* = 0.0242; 72 h p.i. *p* = 0.0045, Unpaired *t*-test). The increase in oxygen free radical is also significantly higher for infection with D39 as it is for infection with all other strains (TIGR4 *p* = 0.0394; R6 *p* = 0.0155; ATCC 49619 *p* = 0.0195, One-way Anova). It should be noted that—as stated earlier—the visual pathology of larvae infected with strain D39 was also worse than that of larvae infected with strains TIGR4 or ATCC 49619. Infection with strain R6 showed no increase in production of oxygen free radicals compared to the control group (1 h p.i. *p* = 0.3951; 24 h pi. *p* = 0.1427; 48 h p.i. *p* = 0.4017; 72 h p.i. *p* = 0.9986, Unpaired *t*-test). This complies with earlier results, as strain R6 was the least virulent strain (Figure 1C). For infection with strain ATCC 49619 (Figure 4C) no increase in oxygen free radicals was observed 1 h p.i. (*p* = 0.2291, Unpaired *t*-test), but there was an increase 24 h p.i. (*p* = 0.0380, Unpaired *t*-test). However, this increase was not observed 48 or 72 h p.i. (48 h p.i. *p* = 0.3521; 72 h p.i. *p* = 0.7146, Unpaired *t*-test). At 24 h p.i. survival of larvae is approx. 33%. This number further lowers to approx. 8% at 48 h p.i. (Figure 1D). This implies most larvae are dying at 24 h p.i., which could explain the temporary increase in oxygen free radicals.

Lastly, in case of infection with heat-killed streptococci (Figure 4A), only directly after infection a difference between infected larvae and control larvae could be observed (1 h p.i. *p* = 0.0494; 24 h p.i. *p* = 0.2665; 48 h p.i. *p* = 0.1941; 72 h p.i. *p* = 0.0679, Unpaired *t*-test).

### Treatment With Amoxicillin or Moxifloxacin Enhanced the Survival of Larvae

**FIGURE 5:**
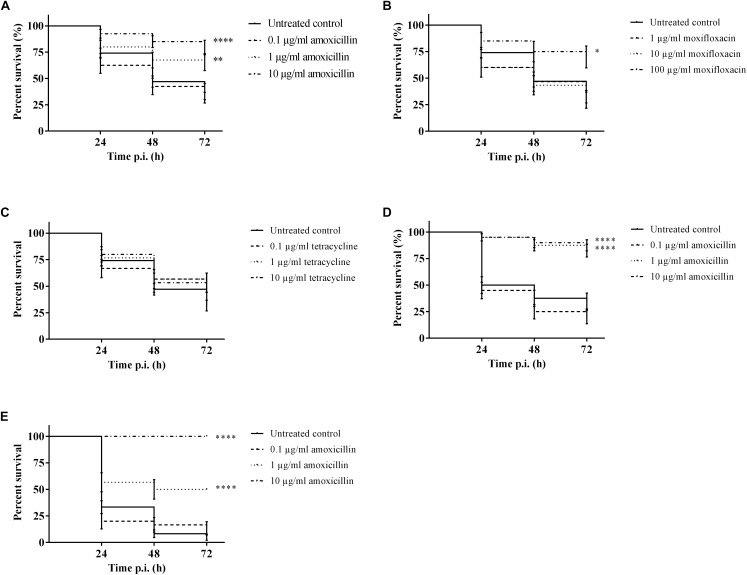
Survival of infected larvae after treatment with different concentrations of amoxicillin, moxifloxacin and tetracycline. **(A)** Survival of larvae treated with amoxicillin after TIGR4/10 infection; **(B)** Survival of larvae treated with moxifloxacin after TIGR4/10 infection; **(C)** Survival of larvae treated with tetracycline after TIGR4/10 infection; **(D)** Survival of larvae treated with amoxicillin after D39 infection; **(E)** Survival of larvae treated with amoxicillin after ATCC 49619 infection. Asterisks represent statistical differences between untreated control groups and treated groups (Survival analysis, *p* ≤ 0.05, ^∗^*p* ≤ 0.05, ^∗∗^*p* ≤ 0.01, ^∗∗∗∗^*p* ≤ 0.0001) (*n* = 10 × 3).

**FIGURE 6:**
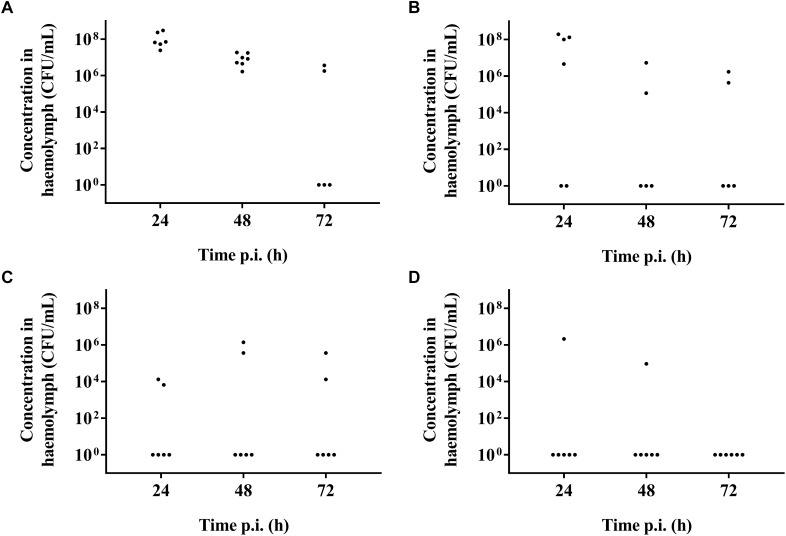
Bacterial burden of larval haemolymph 24, 48, and 72 h post-infection and post-treatment. **(A)** Burden in untreated control larvae; **(B)** Burden after treatment with 0.1 μg/mL amoxicillin; **(C)** Burden after treatment with 1 μg/mL amoxicillin; **(D)** Burden after treatment with 10 μg/mL amoxicillin. Each burden was determined on a pool of 3–5 living larvae (*n* = 2 × 3).

It was possible to treat infected larvae with amoxicillin. For strain TIGR4/10 (Figure 5A), there was no difference between untreated controls and treatment with 0.1 μg/mL amoxicillin (*p* = 0.9725, Survival analysis, Log-rank Mantel-Cox test), but there was a significant difference between untreated controls and treatment with 1 or 10 μg/mL amoxicillin (*p* = 0.0022 and *p* < 0.0001 respectively, Survival analysis, Log-rank Mantel-Cox test). In addition, there was no difference between treatment with 1 or 10 μg/mL amoxicillin (*p* = 0.1179, Survival analysis, Log-rank Mantel-Cox test). The bacterial burden in the haemolymph of larvae infected with strain TIGR4/10 decreased over time for all amoxicillin concentrations used (Figure 6). In concordance with the larval survival results, bacterial burden remained highest in groups treated with the lowest antibiotic concentration.

Secondly, larvae were infected with streptococcal strain D39. While there was no difference between untreated controls and treatment with 0.1 μg/mL amoxicillin for strain D39 (Figure 5D) (*p* = 0.1951, Survival analysis, Log-rank Mantel-Cox test), there was a difference between controls and treatment with 1 or 10 μg/mL amoxicillin (*p* < 0.0001 in both cases, Survival analysis, Log-rank Mantel-Cox test). Also for this strain, there was no difference between treatment with 1 or 10 μg/mL amoxicillin (*p* = 0.5469, Survival analysis, Log-rank Mantel-Cox test). Lastly, larvae were infected with streptococcal strain ATCC 49619 before amoxicillin treatment. Again, there was no difference between untreated controls and treatment with 0.1 μg/mL amoxicillin (Figure 5E) (*p* = 0.8283, Survival analysis, Log-rank Mantel-Cox test), but there was a difference between controls and treatment with 1 or 10 μg/mL amoxicillin (*p* < 0.0001 in both cases, Survival analysis, Log-rank Mantel-Cox test). However, there was also a difference between treatment with 1 or 10 μg/mL amoxicillin (*p* < 0.0001, Survival analysis, Log-rank Mantel-Cox test). This indicates that the dosage highly affected the outcome of infection.

For moxifloxacin (Figure 5B), only the highest concentration of moxifloxacin had an effect on the outcome of infection (*p* = 0.0117, Survival analysis, Log-rank Mantel-Cox test). For 1 and 10 μg/mL no differences in survival compared to the untreated controls could be observed (*p* = 0.6460 and *p* = 0.5833 respectively, Survival analysis, Log-rank Mantel-Cox test). Since TIGR4/10 harbors a tetracycline resistance gene, tetracycline was not expected to influence the course of infection (Figure 5C). Indeed, there was no difference between untreated controls and treatment with 0.1, 1, or 10 μg/mL tetracycline (*p* = 0.1309, *p* = 0.0864, and *p* = 0.0815 respectively, Survival analysis, Log-rank Mantel-Cox test).

## Discussion

In accordance with the “Three R’s” principle (Replacement, Reduction, Refinement), alternative *in vivo* models to vertebrate models have been proposed to lower the costs of drug development experiments and gain data more rapidly (Graham and Prescott, 2015; López Hernández et al., 2015). While these models are often quite different from humans, they do provide valuable information regarding host-microbe interactions and are a useful in-between step in drug development (Ermolaeva and Schumacher, 2014; Trofimov et al., 2017). To study lung infections different animal models without a pulmonary system, such as *C. elegans*, zebrafish, or *G. mellonella*, are currently used (López Hernández et al., 2015). These models are used to investigate interactions with immune cells, and the model of choice is often based on the specific research need of immune cells. For all alternative models, the benefits of low costs, easy handling, lower ethical barriers and fast results often outweigh the differences in immunity between human hosts and these models (López Hernández et al., 2015).

In this study, more in-depth knowledge of a streptococcal infection was acquired. In a previous study using *G. mellonella* larvae, and to the best of our knowledge the only study using streptococci, the effect of a deletion in the polysaccharide capsule of streptococci was determined. The sole outcome parameter of these experiments was the survival of the larvae (Evans and Rozen, 2012). In this study, we consolidated the use of this model to study streptococcal virulence factors by using different streptococcal strains. While there was a difference in survival between serotypes, there was also a difference in survival within the same serotype with or without the polysaccharide capsule. These findings concur with data from other pathogens, such as *Vibrio paraphaemolyticus*, *Streptococcus pyogenes*, and the fungus *C. albicans*, where *G. mellonella* larvae were used to assess the effects of various virulence factors (Tsai et al., 2016; Pérez-Reytor and García, 2018; Rossoni et al., 2018). In our study, streptococcal serotype 19F was the most virulent one, as opposed to most mice models where serotype 19 is commonly less virulent compared to serotypes 2 or 4 (Briles et al., 1992; Chiavolini et al., 2008). However, serotype 19F has been reported previously to be more likely to cause invasive disease in small children compared to in older individuals (Kalin, 1998). This variation could be due to the immaturity of the adaptive immune system of small children. Since adaptive immunity is absent in *G. mellonella* larvae, this could explain the differences in serotype virulence compared to mice. In addition, it is worth noting virulence in mice doesn’t necessarily concur to virulence in humans. Previous research showed clinical isolates of serotype 19F were avirulent in mice, despite having their origin as human disease-causing pathogens (Briles et al., 1992). Furthermore, analogous to vertebrate models and other *G. mellonella* infection models, the inoculum size determines the outcome of infection (MacGowan et al., 2001; Tsai et al., 2016). An inoculum size of 10^6^ CFU/mL was best to observe differences in virulence or to evaluate treatment efficiency. In a previous study, production of oxygen free radicals was investigated and was found to be increased in haemolymph of larvae stimulated with dihydroxyphenylalanine (DOPA) (Slepneva et al., 1999). However, these findings have never been confirmed using bacterial agents. The working mechanism of plasmatocytes and granulocytes is not fully understood, but it is known to involve a burst in oxidative metabolism (Kavanagh and Reeves, 2004). Here, we demonstrated an increase in oxygen free radicals directly after infection with strain TIGR4, yet we did not observe this effect after 24 h of infection or beyond. These data complied with the bacterial burden data, where the burden clearly decreased during the course of infection. This suggests that the haemocytes were partially capable of clearing the infection through the production of oxygen free radicals in a macrophage-like manner rather than oxygen free radicals being produced by the streptococci as a way of battling the host immune system (Slepneva et al., 1999; Bergin et al., 2005). This is confirmed by infecting the larvae with heat-killed streptococci, which are unable to produce hydrogen peroxide. Contrarily, the amount of oxygen free radicals produced during infection with strain ATCC 49619 only increases 24 h p.i., at the time point where most larvae are severely ill or moribund. Hydrogen peroxide is known to be produced by streptococci to combat the immune system and sustain an infection (Preston and Dockrell, 2008; Yesilkaya et al., 2013). For both ATCC 49619 and D39, the increase of oxygen free radicals could be appointed to this. However, if oxygen free radicals were produced by streptococci, haemocytes would start to go into apoptosis and necrosis and numbers would lower, which was not observed (Rai et al., 2015). Nevertheless, numbers might stabilize due to an equal influx of cells to combat the infection and streptococcal killing of cells.

After thoroughly assessing the model, we determined the possibility of antimicrobial treatment. In previous research studies, several therapies against Gram-negative agents, such as *Pseudomonas aeruginosa*, were evaluated using *G. mellonella* larvae. For example, it was recently shown that a cocktail of four novel bacteriophages can cure infections in both mice and wax moth larvae (Forti et al., 2018). For an in-depth overview of evaluation of antibacterial agents using the *G. mellonella* model and a list of pathogens which have already been studied, see the review of Tsai et al. (2016). Treatment of fungal infections has also been proven possible. Recently, the use of probiotic *Lactobacillus* spp. for *C. albicans* infections has been studied and proven effective (Santos et al., 2018). Furthermore, in the first 6 months of 2018 alone, 5 novel research studies were published evaluating both prophylactic and therapeutic treatment options for *C. albicans* in *G. mellonella*—showing a clear interest and application for this model (Liu et al., 2017; Borowiecki et al., 2018; Chupáčová et al., 2018; Gu et al., 2018; Staniszewska et al., 2018). Here, we assessed the functionality of 2 standard antibiotics, amoxicillin and moxifloxacin. Amoxicillin is a bactericidal beta-lactam antibiotic commonly used in the first-line treatment of lower pulmonary infections in both children and adults. Moxifloxacin is a fluoroquinolone used when amoxicillin is contra-indicated due to a penicillin allergy in adults (Belgische Commissie voor de Coördinatie van het Antibioticabeleid, 2012). We observed a clear dose-response effect in our *in vivo* model for both amoxicillin and moxifloxacin and observed a decrease in bacterial burden after amoxicillin usage compared to a non-treated group.

There are several limitations to the wax moth model. Firstly, in contrast to mammalian models, *G. mellonella* larvae do not possess an adaptive immune system (Wojda, 2017). However, the innate immune system is the first-line defense in human lungs through the presence of alveolar macrophages (Marriott and Dockrell, 2007; Camberlein et al., 2015). Therefore, the larval model – with its phagocytic haemocytes – can be used as a substitution for more complex vertebrate models. Larval haemocytes possess a similar mechanism for phagocytosis compared to human macrophages, also using reactive oxygen species through activation of the NADPH oxidase complex. In this larval complex, immunologically related proteins to human p47^phox^ and p67^phox^ have been observed (Bergin et al., 2005). While the differences in immunity between *G. mellonella* larvae and vertebrates limit the possibility of replacing vertebrate *in vivo* models, this model can be used as an in-between step between *in vitro* and complex *in vivo* models (Tsai et al., 2016).

This model has previously been evaluated for its use in toxicity studies and antimicrobial susceptibility testing using standard antibiotics and several Gram-positive and Gram-negative strains. A correlation between toxicity in wax moth larvae and rodents could be observed. Furthermore, the therapeutic doses for human use of the tested antibiotics could be translated to the doses in larvae (Ignasiak and Maxwell, 2017). Also, the model is currently being used for the evaluation of potential novel antimicrobial strategies against pathogens such as *C. albicans*, *Burkholderia cenocepacia*, *Porphyromonas gingivalis*, and *Klebsiella* *pneumoniae* (dos Santos et al., 2017; Van den Driessche et al., 2017; Nath et al., 2018; Staniszewska et al., 2018). The implementation of this model leads to a lower number of compounds going into further *in vivo* trials, thereby reducing the number of vertebrates (Ignasiak and Maxwell, 2017). While the determination of streptococcal bacterial burden currently requires larval death, bioluminescence could be a valuable addition to the model. Use of bioluminescence for *in vivo* imaging in *G. mellonella* has been previously assessed using *lux* tagged promotors of virulence factors in *L. monocytogenes* infections (Joyce and Gahan, 2010). In fungal infections, bioluminescence has been used to study *C. albicans* burden both *ex vivo* and *in vivo* (Delarze et al., 2015). More recently, bioluminescence has been applied to study *in vivo* infections with *Mycobacterium abscessus* (Meir et al., 2018).

## Conclusion

In conclusion, our results strengthen the idea of implementing the *G. mellonella* model in the drug discovery process on a standardized basis. It is possible to evaluate host parameters, such as haemocyte count and production of oxygen free radicals, and to monitor bacterial burden during the course of infection. This model could provide valuable information regarding potential novel antimicrobial compounds, without the use of expensive and ethically challenging vertebrate models. While this model cannot replace the vertebrate models currently used, it can be a valuable addition in the transition from *in vitro* to *in vivo* data.

## Data Availability Statement

The raw data supporting the conclusions of this manuscript will be made available by the authors, without undue reservation, to any qualified researcher.

## Author Contributions

FC, BV, and PC conceived the presented idea. FC carried out the experiments. ET, JA, BV, DC, PD, and PC contributed to interpretation of results. FC wrote the manuscript with support of ET, JA, BV, DB, LM, and GC. PD and PC helped supervise the project. All authors contributed to manuscript revision, read, and approved the submitted version.

## Conflict of Interest Statement

The authors declare that the research was conducted in the absence of any commercial or financial relationships that could be construed as a potential conflict of interest.
