# Supplementary material for: Optimization and Characterization of a Galleria mellonella Larval Infection Model for Virulence Studies and the Evaluation of Therapeutics Against Streptococcus pneumoniae
Source: Front Microbiol. 2019 Feb 21;10:311. doi: 10.3389/fmicb.2019.00311 (PMC6394149; doi:10.3389/fmicb.2019.00311)
Supplement: Supplementary file 1 [file Image_1.pdf]

SUPPLEMENTARY DATA

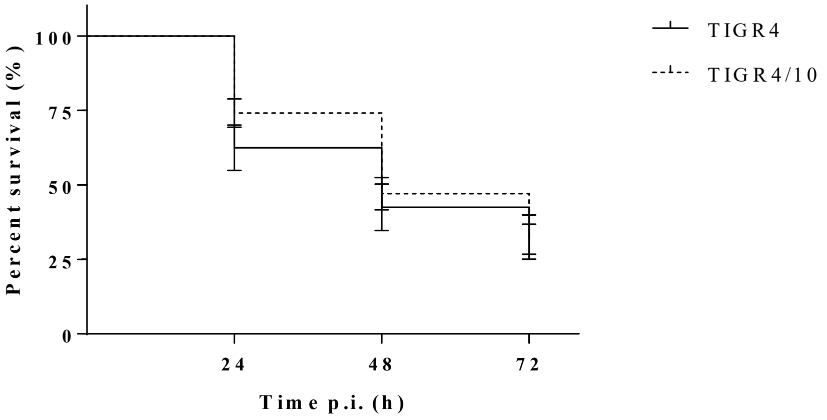

Supplementary Figure 1: Comparison of larval survival between TIGR4 and TIGR4/10 infection. There was no statistical difference between survival of TIGR4 infection compared to TIGR4/10 infection ($p = 0.8658$, Log-rank (Mantel-Cox) test). ($n = 10 \times 4$)
